# Barriers to Cervical Cancer Screening and Satisfaction with Self-Sampling among Black Women in Michigan: a Mixed Methods Study

**DOI:** 10.18103/mra.v12i4.5209

**Published:** 2024-04-26

**Authors:** Elizabeth Haro, Emma A. Butcher, Martha L Alves, Christelle El Khoury, Alexandra Vinson, Diane M Harper

**Affiliations:** 1 Department of Family Medicine, University of Michigan, Ann Arbor, MI; 2 Department of Learning Health Sciences, University of Michigan, Ann Arbor, MI; 3 Department of Obstetrics and Gynecology, University of Michigan, Ann Arbor, MI; 4 Department of Women’s and Gender Studies, University of Michigan, Ann Arbor, MI

**Keywords:** mixed methods, HINTS, Theoretical Domain Framework, Barriers to cervical cancer screening, patient preferences for physician characteristics, speculum exam, self-sampling for cervical cancer screening, African American, Black, women, under-screened, adult, HPV

## Abstract

**Background.:**

In recent years, cervical cancer screening among Black women in the United States has declined, followed by increased incidence and mortality. We aim to evaluate the individual, sociocultural, and structural barriers to cervical cancer screening in relationship to the exam technique barriers.

**Methods.:**

Participants received cervical cancer self-screening kits in the mail. They returned their samples and a quantitative survey developed from the Health Information National Trends Survey (HINTS) modules designed to address the known individual, sociocultural, and structural barriers to screening. We established the fourteen attributes of cervical cancer screening techniques from prior work. Participants then shared their experiences in a semi-structured qualitative interview informed by the Theoretical Domains Framework (TDF) to explore the answers to the survey questions. We coded themes from the interviews. Women were grouped as younger (30–45 years) and older (46–65 years).

**Results.:**

Of the 41 women completing the study, 21 were in the younger age group (mean 37.3, SD 4.7), and 20 were in the older age group (56.5 (5.5)). All participants self-identified as African American/Black and were due for cervical cancer screening. Women indicated that individual, sociocultural, and structural barriers influenced their cervical cancer screening, but the most significant barrier was the speculum-based technique itself. Three positive attributes and eight negative attributes significantly differed by screening technique, favoring the self-screening technique.

**Conclusions.:**

The self-screening technique for screening for cervical cancer is feasible and acceptable to this group of Black women.

## Introduction

Cervical cancer prevention in the United States starts with screening under the assumption that if more women were screened, more women would be prevented from having cervical cancer. Over time access to screening was different for women of color, often being done in isolation of their total health care and done under systemic prejudice.

In 1973, 59.9% of Black women had screened for cervical cancer within the past two years, as recorded by the National Health Interview Survey (NHIS), significantly lower than White women (64.2%). But by 1985, across the US, this trend reversed, and more Black women were screened than White women (70.5% vs 64.1%).^1^ This increase was partly due to the Black Church’s role as a locus for delivering social and preventive health.^2–6^ This trend continued until 2019, when Black women’s screening rate dropped below White women’s.^7^ Despite screening efforts, Black women continue to have a higher incidence of cervical cancer than White women with the disease diagnosed at a later stage, with a lower survival rate.^8,9^

The documented barriers to screening for Black women in the US include individual, sociocultural, and structural factors.^10^ Individual barriers include competing priorities (no time to go), financial barriers (no health insurance, no transportation, no childcare), discrimination at the clinic, fear of the results, and a history of trauma.^11–14^ The sociocultural facilitators and barriers include religiosity (facilitator), social stigma, and mistrust (barriers) in the healthcare system.^14,15^ The structural barriers include a lack of access to race-concordant health care.^16^

In Michigan, fewer Black women (85%) than White women (92%) have ever had a cervical cancer screen, and 28% are not up to date on their screening.^17^ This work aims to evaluate the barriers to cervical cancer screening among Black women residing in southeast Michigan and investigate how a vaginal self-sampling collection technique may change screening practices.

## Methods

This mixed methods study design synthesizes a purposeful survey with brief semi-structured interviews to discern how previously published inequity barriers to cervical cancer screening may apply to urban/suburban Black women in Michigan. Our qualitative methods are reported per COREQ guidelines.^18^

### RECRUITMENT.

Members of the research team are all women and have experience and training in public health, medicine, social work, sociology, and qualitative methods. We partnered with an African-American community non-profit organization (Detroit, MI), a federally qualified health center (Flint, MI), and a university research recruitment registry (Ann Arbor, MI) to recruit women who had not been screened for cervical cancer within the past three years. All collaborators advertised our study opportunity and requested that interested women contact our study team. Recruitment also included snowball sampling from contacts referred by existing participants.

### POPULATION.

Eligible women were those 30–65 years of age, who self-identified as Black, were due for cervical cancer screening, were not pregnant, did not use a pessary, and had not had a hysterectomy.

Women meeting the study inclusion criteria were enrolled in sequence from a community sample to a pre-defined age stratification of at least 20 women in each age group: 30–45 and 46–65 years old, representing pre- and post-menopausal women. Recruitment was entirely remote and took place between November 2020 and May 2021. Of those women who contacted the research team, two declined to participate, 14 agreed to participate but did not complete the study, and 41 agreed to participate and completed the study.

### SURVEY QUESTIONS.

We used the National Cancer Institute (NCI)’s Health Information National Trends Survey (HINTS) for survey design, including demographics, cervical cancer, cancer perceptions and knowledge, health status, patient-provider communication, and risk perceptions modules.^19^ These questions provide insight into individual, sociocultural, and structural barriers. HINTS limited the barrier questions to eleven pre-defined reasons. As such, a “none” response only indicates not any of the pre-defined reasons.

### INTERVIEW QUESTIONS AND DATA COLLECTION.

Using an interpretive description approach, the brief semi-structured interview sought to understand the experience of cervical cancer screening in traditional physician-led settings and the self-screening protocol to produce actionable insights that inform changes in healthcare delivery.^20^ We used the Theoretical Domains Framework of Behavioral Change (TDF) to develop our interview guide that allowed women to share past experiences most salient to them about the cervical cancer screening process.^21,22^ The interview guide was not pilot-tested but was grounded in the TDF. One of two researchers (EKH, CEK) interviewed each woman by phone. The semi-structured interview guide allowed women to describe their experiences in response to open-ended questions. Participants were interviewed only one time.

### SELF-SAMPLING COLLECTION TECHNIQUE.

We mailed all participants a package containing a paper survey, a vaginal self-collection kit (HerSwab^®^, Toronto, ON, CA), instructions on how to use the vaginal kit, and a return pre-paid postage box for the kit’s return. We conducted interviews after the women returned the collection kits.

### ANALYSES

#### Quantitative Analyses.

The study was powered a priori to show a clinically significant difference of one point per attribute on the Likert 1–5 rating scales if at least 36 women enrolled.^23,24^ Power is 90% with a type I error (two-sided alpha) of 0.05, correcting for 14 multiple comparisons assuming a standard deviation of one unit and a between-group correlation of 0.1 unit. We completed the quantitative analyses with descriptive statistics, t-tests, one-way analysis of variance, and the Mann-Whitney-U test for non-parametric testing. We applied the Bonferroni correction for multiple comparisons for each set of comparators. We provide all p-values for the appropriate Bonferroni correction. We used Statistica v14.0 software.^25^

#### Qualitative Analyses.

The brief one-on-one interviews were audio-recorded with the consent of the participants, transcribed, and checked for accuracy by research staff before analysis. Three participants declined to be recorded, and notes were taken by the interviewer instead. On average, the interviews lasted 8:27 minutes (range 3:49 to 24:22 minutes), and transcripts were not returned to interviewees for review or member checking.

Three female researchers (EKH, EA, MA) analyzed the interview transcripts through inductive open coding and discussion. Frequent discussions between coders allowed research team members to achieve and maintain consensus on the meaning and application of codes. In the interpretive description tradition, saturation is not a standard for the quality or completeness of data collection.^20^ However, we designed a focused interview guide and drew the participants from a relatively homogenous population. This indicates that the sample size was likely appropriate for identifying patterns in experience in this sample, with potential transferability to other similar populations beyond the study sample.^26^

The qualitative coding process produced three major themes and four minor themes that help us describe the experience of participants in using the self-sampling collection kit and how they contrast this experience with a traditional pelvic exam. Major themes included convenience, contrast between testing methods, comfort, and self-empowerment. Minor themes included impact on future screening, accessible answers to screening technique questions, perceptions about the accuracy of the screening technique, and feeling shame at having to undergo a clinician-directed speculum exam. We include illustrative quotes to support each finding, using ages and pseudonyms to protect anonymity.

## Results

We present the results by section domains.

### POPULATION DESCRIPTORS

Of the 41 women completing the study, 21 were in the younger age group (mean 37.3, SD 4.7), and 20 were in the older age group (56.5 (5.5)). All self-identified as African American or Black. All women lived in an urban/suburban location, and 60% were employed full-time or part-time (Table 1). Most women (70%) had a routine healthcare check-up within the last two years. No women 30–45 years old, and only 85% of the 46–65-year-olds, were menopausal (Supplemental Tables 1 and 4).

The differences in women’s preferences for specific physician characteristics were significant by their age and insurance status. Younger women disagreed more often that their physician needs to be of the same religion/culture as they are compared to older women (2.0 (0.9) vs. 2.6 (0.6), p<0.05). In addition, those women who had Medicare or Medicaid insurance disagreed more than women with private insurance that “the gender of the physician impacts my comfort level and willingness to participate in a pelvic exam” (2.4 (1.4) vs. 3.4 (1.4), p<0.05).

Women with higher educational achievement disagreed significantly more than those with lower educational achievement that they avoided a pelvic exam because of religious/cultural reasons (1.2 (0.4) vs. 1.6 (0.8), p<0.05). (Supplementary Table 2).

### INDIVIDUAL BARRIERS

When asked to choose from a defined list of individual barriers, 70% of women selected none of the listed barriers, while 15% chose a single barrier and 15% chose multiple barriers. Of single barrier choices, having no health insurance coverage for screening was the most common, followed by no time to go for screening, no transportation, not knowing *that* she needed screening, and being scared of the results. Of the women choosing multiple reasons, 100% chose “not knowing *when* she needed to go for screening.” Half of the women also chose “no health insurance coverage for screening” or “no time to get screened.”

### EXAM TECHNIQUE

**Figure 1.**
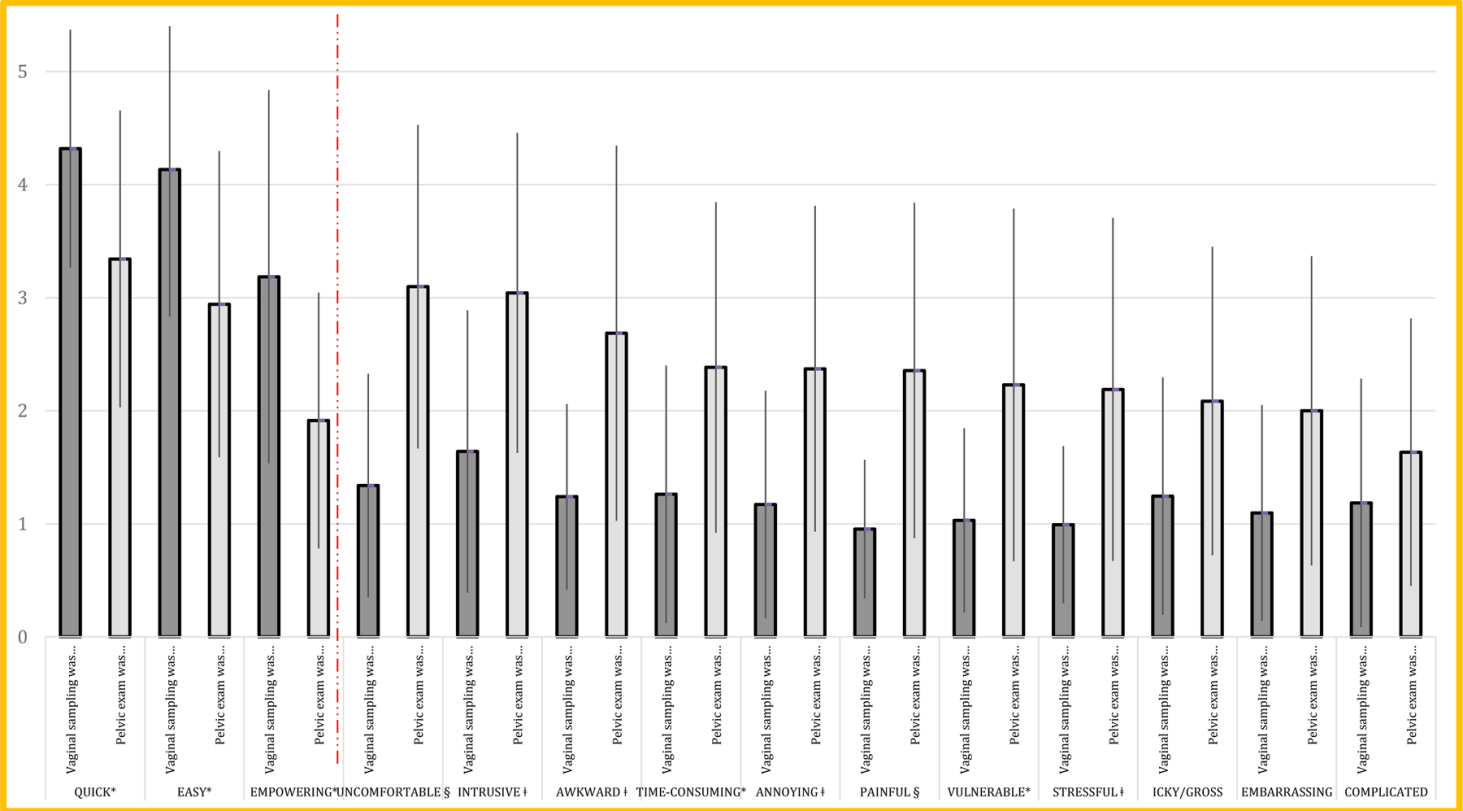
Overall Exam Techniques Comparisons Three positive attributes of screening techniques are on the left of the red line. Eleven negative attributes are on the right. The vaginal self-sampling technique to pelvic exam technique is significantly different after correcting for multiple comparisons: *p<0.05, ǂp<0.01, §p<0.001

The three positive attributes of any cervical cancer screening technique are being “*quick*,” “*easy,*” and “*empowering*.” Vaginal self-screening ranked significantly higher for these attributes than the speculum exam technique (Figure 1). Similarly, eight negative attributes of a screening technique were minimized considerably by the vaginal self-screening technique. Significant were the differences in “*pain,” “uncomfortable,” intrusive,” “awkward,” “time-consuming,” “annoying,”“ stressful,” and “vulnerable.”*

Among the negative attributes of the pelvic exam, the pelvic exam as “*uncomfortable*” ranked the worst. Nine women (22%) ranked the pelvic exam as uncomfortable at its maximal score. When asked to rank all fourteen attributes of the collection techniques, these women rated all of the negative attributes of the pelvic exam very poorly, and all nine indicated a future intent to screen with self-sampling instead of the speculum exam.

**Figure 2.**
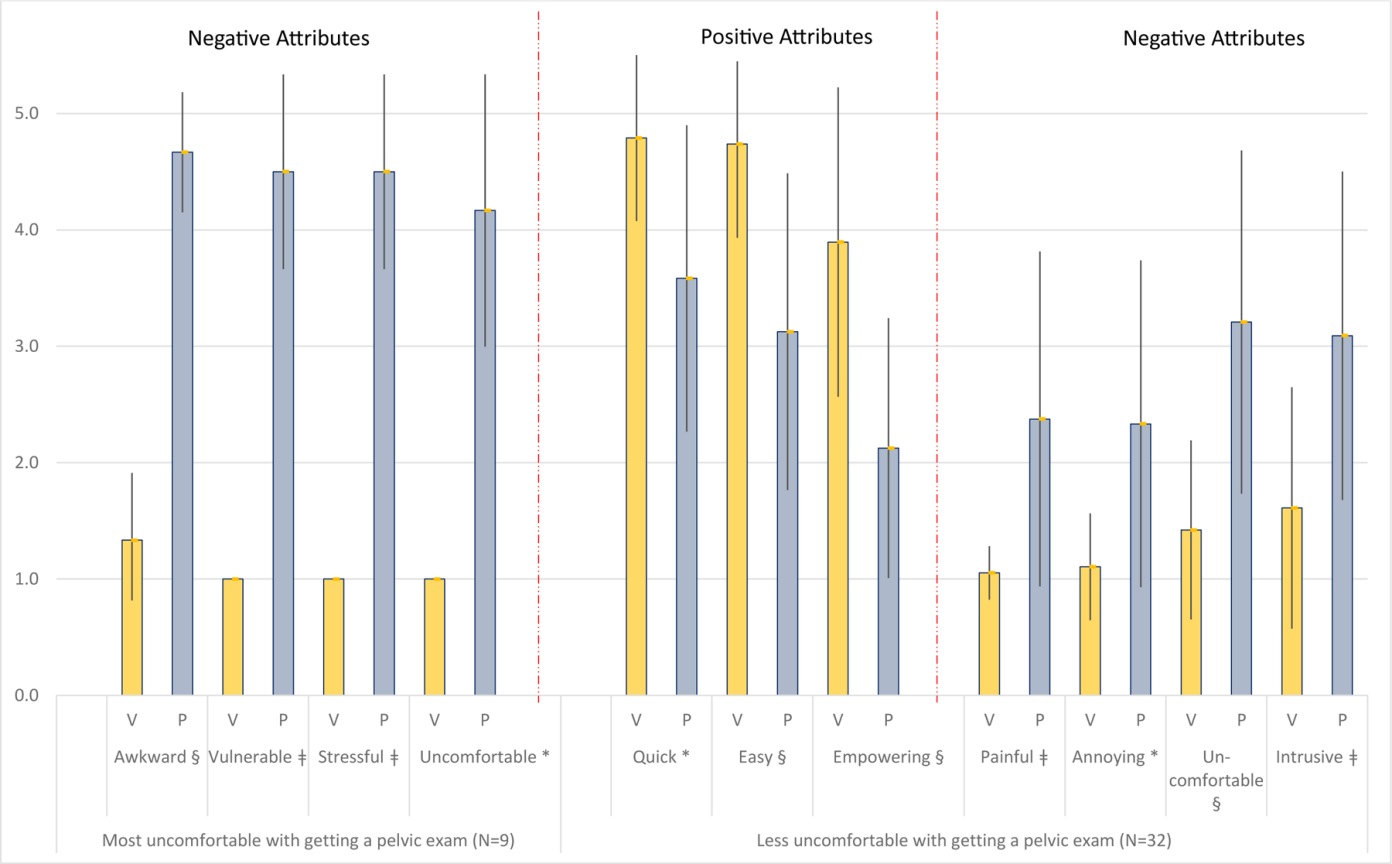
The mean (SD) of the attributes of the collection techniques by her comfort/embarrassment with getting a pelvic exam Exam techniques are V=vaginal self-sampling and P=pelvic exam technique. Exam technique rankings differ by agreement/disagreement with the concept that the pelvic exam is uncomfortable or embarrassing. *p<0.05, ǂp<0.01, §p<0.001 corrected for multiple comparisons. The attributes shown were statistically significant when dividing the population by embarrassment/uncomfortableness with the pelvic exam.

The remaining women (N=32) who had less uncomfortableness with the pelvic exam ranked the positive attributes of the self-screening technique much higher than the speculum exam technique and the negative attributes much lower (Figure 2). Most significantly, the vaginal self-sampling technique was less “*painful*” than the pelvic exam, as well as less “*annoying*,” “*uncomfortable*,” and “*intrusive*.” Nearly all of the women in this group indicated a preference for self-screening (58%) or indifference to the technique (38%) for future cervical cancer screening. In addition, negative attributes of the speculum exam technique vary by women’s educational achievement and need for the same gender for the pelvic exam (Supplemental Table 3).

### QUALITATIVE ANALYSES

When asked which technique they preferred, 82.9% (34/41) chose self-sampling over a pelvic exam. For example, Erica, age 35, was part of this majority, saying, “*The kit was 100% easier than having to come in and get a speculum… I would choose to do this any day over having to come in for a Pap smear*.” Donna, age 59, said, “*I much prefer the kit. Oh my gosh, one thousand percent,*” and Amber, age 38, stated, “*I would rather do that over going to the doctor and getting a Pap smear.*”

Veronica, age 49, echoed this, saying, “I would much rather have the kit,” and Brittany, age 46, described self-sampling as “*It’s like a party, it was so easy, it was not intrusive… If I could do this all the time, I would.*”

Focusing on their experience using the self-sampling kits at home, inductive analysis of the semi-structured interview questions revealed several key themes.

#### Theme 1. Convenience

##### Convenience of the self-sampling technique *for screening location*.

The convenience of self-sampling was the most prominent theme, emerging from 93% of the interviews (38/41). Many participants described the convenience of home-based screening in terms of eliminating barriers to traditional in-office screening. These included scheduling appointments and arranging transportation, childcare, and time off from work. For example, Aaliyah, age 35, said, “*It’s inconvenient to have to go to the doctors… You got to make the appointment, possibly miss work, sit in the lobby… It’s just a lot that goes with it.*” Camille, age 41, expressed a similar sentiment, listing several barriers that can make it challenging to attend the in-person screening: “*You don’t got no gas money, you don’t got this or that, I got to work.*” Andrea, age 52, identified an additional barrier: “*If you got kids and you don’t want your kids at the doctors’ office, they can’t be in the room with you, so you got to find a babysitter.*”

##### Convenience *for time flexibility*.

Flexibility was a key convenience aspect for many, including Michaela, age 37, who said, “*It’s just the convenience of being able to do it on my own time. And not having to take extra time out of my day. Especially having two small children, just the convenience of it is really nice.*” Participants appreciated being able to screen when it best fit their schedule. For example, Michaela also added, “*It was just super convenient that I didn’t have to make an appointment, take time out of my workday, or go anywhere else. I could do it in the evening once I got home. It was a good time for me.*” This was also true for Mariah, age 38, who said, “*It’s hard to move your schedule to figure out school, and then some people have to work from home. So it’s more easy and accessible where you can like just say, ‘Okay, I got like 15 minutes.’ You know, I can go ahead and do it.*” Jordan, age 31, summed up home-based screening as: “*You have the convenience to get the same results on your time.*”

#### Theme 2. Uncomfortable/Comfortable Attribute of exam technique

##### Painful discomfort with the clinician-directed speculum exam.

Comfort with the collection technique was the next most prominent theme, emerging from 78% of the interviews (32/41). For many, this theme was described in the context of past experiences with traditional clinician-directed screening. Words representing all ranges (from a little to a lot), such as “*embarrassing,*” “*uncomfortable,*” “*painful*,” “*ashamed,*” and “*violated,*” were used by participants when talking about these past experiences. For example, Veronica, age 49, described her discomfort with the physical exam: “*When you are getting an exam at the doctor, it’s really uncomfortable, and it can be kind of degrading because somebody is actually, you know, going in you that you don’t even know.*” This was echoed by Michelle, age 43, who described her concerns with the sensitivity of the physical exam and physical discomfort with the procedure itself: “*Even if it is something you are fairly comfortable with, like with your body, it is still not great. I can’t say that I enjoy having to disrobe and having someone that I don’t know touch me very intimately. And like, the examination process is pretty uncomfortable, in terms of getting like the Pap smear because they are kind of like poking you with a swab.”*

##### Easy comfort with private screening location.

Many participants highlighted how the comfort of the self-sampling technique improved their experience with screening. In various ways, screening at home allowed them to avoid the discomforts they associated with traditional clinician-directed screening. Instead, home-based screening offered participants privacy. Veronica said, *“I feel much more comfortable doing it myself in the privacy of my own home.*” After she characterized a cervical cancer screen as “*Just a lot of things about it that are not great,”* Michelle went on to say that self-sampling’s ability *“To eliminate all of that is actually a huge improvement.*” Andrea specifically liked how “*At home, you don’t have an audience,*” and Brittany said self-sampling was “*A test that can provide the results in the least intrusive way*.” Harkening back to her description of having to “*suffer*” through traditional speculum screening, she added, *“Anything that reduces that sort of discomfort is a welcome development, I think.*”

#### Theme 3: Empowerment through Self-Sampling

Lastly, a theme centering on *empowerment* was also present in 22% of the interviews (9/41). Participants talked about how, with self-sampling, they valued playing a more active role in their own healthcare. This was the case for Amber, age 38, who said, “*I like the fact that I can do it on my own. That kind of gives me, you know, some type of- what’s the word I’m looking for well, maybe I can use empowerment. You know it’s my health and I like that I’m doing it, you know what I mean? Versus them doing it. It feels good that I can do something like that for myself health-wise.”* For others, it was having the act of sample collection in their own hands that was most salient. Veronica, age 49, said “*for example, like how with self-sampling, It’s under your control.*” Andrea, age 52, added, “*When I did that for myself, I was like, okay, this could be something cool.*”

##### The agency that came from being *offered a choice*

was also seen as contributing to feelings of empowerment. For example, Dawn, age 63, said, “*It was so empowering to be able to have those kinds of choices. It’s like, wow, this is great. I can, you know, choose which one I want and then, you know, I can pick the time and when it’s more convenient for me. I’m just more relaxed in my own home so I think it’s better.*” Still, other participants mentioned that they felt empowered because of self-sampling’s perceived benefit for a larger community of women. Michelle, age 43, reported this feeling by saying, “*I would recommend it really highly, anything that empowers women to take charge of their health and stay aware of screening things like that, and to do it themselves.*” Dawn also added, “*Wouldn’t this be great if we can be a part of something revolutionary like this to help women in the future?*”

##### Minor Theme 1. Impact on Future Screening

In the final part of the interview, we asked participants how they thought access to self-sampling could impact their future screening participation. The majority of responses indicated that such a choice would positively impact future screening intent (53.7% (22/41) ) or not matter (41.5% (17/41). No one indicated a negative impact. Jasmine, age 30, said, “*I feel like this type of screening material kind of bridges that gap between like, okay, knowing I need to get this done, but I am not really comfortable with someone seeing that, but now I can still get the screening and be comfortable about it, and do it correctly.*”

##### Minor Theme 2. Accessible answers to screening technique questions

While most participants preferred self-screening at home, some women (11/41) indicated they would either request self-screening in the office or had no preference between screening techniques. The ability to ask questions and ensure the sample was correctly collected drove responses for these women. For example, those who preferred to continue going to the office for screening would still choose self-screening. This was the case for Mariah, age 38, who said, “*I would say in a clinic where, you know, like if you are having some problems, you can ask questions… Doing it yourself but just have them as an assistance where if you need that extra help.*”

##### Minor theme 3. Opportunities for education about the accuracy of the screening technique

For a small number of participants (4/41, 9.8%), clinician-directed screening was perceived as a more accurate way to conduct screening for an average-risk asymptomatic woman. For example, Rhonda, age 61, said, “*It feels a lot more thorough if I go into the doctor’s office… For the pelvic exam, they are looking at other things and you can’t get that at home.*” Similarly, Dawn, age 63, stated, “*It just seems like at the doctor’s office they- it seems like they would get a better sample… like it goes in deeper, you know? And so, it just seems like it would be less of a chance of anything being missed than at home.*” All of these statements are medically incorrect and call for education.

##### Minor theme 4. Feeling shame at having to undergo the speculum exam technique

Two women named a theme that others hinted at through their conversational tone: having to tolerate the deprecating speculum exam technique. Brittany, age 46, summed up the experience as **resignation without choice**: “*We all go to the GYN, we all have to suffer through the same things… We just do what we got to do for our health.*” Dawn, age 63, said, “*I think when it comes to making sure that it’s done right, I think that even though it’s embarrassing, I would rather have my healthcare provider do it to make sure it’s done right. So I’ll* ***just bear the shame you know*** *for like a minute or two in order for it to be done correctly.*”

## Discussion

The speculum exam is steeped in institutional mistrust, abuse, and trauma of past generations of Black women.^27,28^ Despite the majority of the respondents indicating no pre-specified barrier to cervical cancer screening, we showed that, when asked about detailed attributes of screening after using the self-sampling kits, all women ranked the speculum exam technique as a significant barrier precisely because it took place in the clinic setting by appointment and because it posed concerns about privacy and intimacy. It is the recognition that the speculum-based pelvic exam caused such physical/spiritual and emotional expressions in this population that a call out for other options for cervical cancer screening is so important. Other barriers exist, but we focused on their post-kit experiences compared to the speculum exam technique.

We showed that the self-screening technique significantly reduced the perceived pain of the screening, allowed most women to trust themselves to screen, and removed the resignation of embarrassment.

African American women residing in the urban/suburban area of southeast Michigan continued to share known *individual* barriers to cervical cancer screening, as has been described in prior work.^29,30^ Specifically, our population described not knowing when to be screened, having no time or transportation, and perceived insufficient insurance to cover screening exams as single or part of a bundled set of reasons not to screen. However, all of these barriers can be lessened by the ease, convenience, and empowerment of the cervical cancer self-sampling technique our population showed was preferable to a traditional speculum exam technique.

A small portion (5%) of our population also expressed the fear of the results as both a single *individual* barrier and one of many barriers to screening. Fear is acknowledged in prior work as an aspect of fatalism that can be overcome with education.^13^

While prior work in African American churches indicates *sociocultural* barriers to screening seeded in the woman’s religiosity (social stigma around the association between sexual activity and cervical cancer screening),^10,15^ our work shows that higher educational attainment may mitigate the influence of religion. Specifically, we showed that Black women with higher educational attainment disagreed that they would avoid a pelvic exam more because of their religious beliefs.

Unlike prior work that indicates a *structural* barrier of lacking physicians of the same race/ethnicity as the women,^16^ our population instead was influenced by the *gender* of her physician, not the race, showing that the speculum exam technique is more annoying and makes her feel more vulnerable when the gender of the person doing her exam is essential to her. This aligns with prior research that shows that gender plays a more decisive role than race in matching physician and patient attributes for healthcare delivery.^31^

While others have shown that Black women prefer the convenience, privacy, and lack of cost (in terms of transportation, time, appointment, childcare, and work schedule) of the self-sampling collection techniques,^32,33^ ours is the first to document the direct comparison of fourteen attributes of screening techniques that included the speculum-based exam technique.

Our work provides the intense response via quotations that Black women offered about the cervical cancer screening process that interwove the individual, sociocultural, and structural barriers with very personal narratives supporting the use of a self-screening technique for cervical cancer screening seen in the major and minor themes. Relieving the sense of shame and resignation that accompanied an action to improve her health is immediately necessary. Restoring the sense of empowerment to every woman needs to be the public health and individual care goal to reduce and eliminate cervical cancer.

## Strengths and Limitations.

This work offers a strength no other prior analysis has. All women who participated in this study had the opportunity to use a self-sampling kit with immediate access to the research staff should any questions arise. They then shared their experience via the one-on-one interview, providing insights into their experience that the quantitative survey could not capture. Our mixed method design allowed us to evaluate new screening techniques that the women experienced in the context of prior known barriers to cervical cancer screening. Our work supports the feasibility and acceptability of self-screening for cervical cancer screening in this population of Black women.

Two aspects of the research process pose potential limitations to the understanding achieved by this study. First, although the research team was all women, which may have made it easier for participants to speak candidly about past experiences with pelvic exams and cervical cancer screening, no members of the research team were Black. This may have shaped the level of candor and how participants expressed their experiences as Black women with cervical cancer screening and gynecological care. Second, while trusted community organizations facilitated recruitment, which may have helped to ease the relationship between individuals interacting with institutional researchers, there may have been generalized institutional mistrust that created a selection effect among participants.

In addition, we acknowledge that 14 women who consented to the study did not return their kits or questionnaires after we made the three attempts allowed by the IRB consent. When there was no response, the research team left a text message or voicemail as the preferred method of communication. There could have been many reasons for this loss of follow-up that had nothing to do with the study. Others may not have found the idea of using self-sampling palatable. This limitation indicates our report may be biased towards those who ranked the kit use satisfactorily. On the positive side of participation, the study occurred during the COVID-19 pandemic when in-office exams were unavailable, potentially enhancing the opportunity to try new techniques. Nevertheless, the research team found that women who did participate spoke openly and in specific terms about their experiences. Our work shows that evidence-based screening techniques can ease the discomfort described by participants and promote self-empowerment through increased control with cervical cancer screening.

Another possible limitation of our study is that there is ethnic diversity among Black women.^34^ Future opportunities exist to investigate how Black women from different ethnic backgrounds receive self-sampling as a cervical cancer screening option and to explore barriers and facilitators of traditional speculum exams.

## Conclusions

Our study shows that Black women positively welcomed a vaginal self-screening technique for cervical cancer screening in an urban/suburban area of Southeast Michigan.

## Supplementary Material

1

## Figures and Tables

**Table 1.** Population Descriptors by Employment Status

	Employment Status				TOTAL	
	Full or Part Time		Unemployed			
**Location**	**N**	**%**	**N**	**%**	**N**	**%**
Rural	0	0%	0	0%	0	0%
Small Town	0	0%	0	0%	0	0%
Mid-size Town	10	42%	8	50%	18	44%
Suburbs	9	38%	1	6%	10	24%
Large Town or City	5	21%	7	44%	13	32%
**Education** *						
Less than 8th grade	0	0%	0	0%	0	0%
8–11th grade	1	4%	4	25%	6	15%
HS graduate or GED	3	13%	5	31%	8	20%
Vocational/technical school	11	46%	4	25%	15	37%
Some college	5	21%	2	13%	7	17%
College Graduate	4	17%	1	6%	5	12%
Graduate						
**Employment**						
Full time	16	67%	0	0%	16	40%
Part-time	8	33%	0	0%	8	20%
Unemployed for less than one year	0	0%	6	38%	6	15%
Unemployed for more than one year	0	0%	1	6%	1	3%
Homemaker/Caretaker	0	0%	3	19%	3	8%
Student	0	0%	1	6%	1	3%
Retired	0	0%	2	13%	2	5%
Disabled	0	0%	3	19%	3	8%
**Income** **						
Living comfortably	10	43%	1	7%	12	31%
Getting by	9	39%	7	47%	16	41%
Finding it difficult to get by	3	13%	2	13%	5	13%
Finding it very difficult to get by	1	4%	5	33%	6	15%
**Insurance Status** *						
Employer-based	10	50%	1	7%	11	31%
Purchased on own	0	0%	1	7%	1	3%
Medicaid	7	35%	10	67%	18	50%
Medicare	2	10%	2	13%	4	11%
None	1	5%	1	7%	2	6%
**Health Status** **						
Excellent	3	13%	0	0%	4	10%
Very good	10	42%	2	13%	12	29%
Good	9	38%	7	44%	16	39%
Fair	2	8%	7	44%	9	22%
**How long since your last routine health check-up**						
Within past year	9	38%	6	40%	16	40%
1–2 years	9	38%	3	20%	12	30%
3–5 years	6	25%	4	27%	10	25%
More than five years	0	0%	1	7%	1	3%
Unsure	0	0%	1	7%	1	3%

* p<0.05

** p<0.01 by MWU tests

Full- or part-time employed women had higher educational achievement (Z adjusted = −2.35, p<0.05) than unemployed women. Likewise, women with full/part-time employment had higher incomes, private health insurance, and higher self-rated health status than those not employed (Z adjusted=2.75, p<0.01, Z-adjusted=1.98, p<0.05, Z-adjusted=3.12, p<0.01, respectively).
