# Supplementary material for: Barriers to Cervical Cancer Screening and Satisfaction with Self-Sampling among Black Women in Michigan: a Mixed Methods Study
Source: Med Res Arch. Author manuscript; Available in PMC 2024 May 30. (PMC11138408; doi:10.18103/mra.v12i4.5209)
Supplement: 1 [file NIHMS1986026-supplement-1.pdf]

SUPPLEMENTARY MATERIAL

Supplemental Table 1. Reproductive descriptors by birth control use

	Birth Control				Total	
	None		Any			
	**N**	**%**	**N**	**%**	**N**	**%**
**Current Birth Control**						
Male condom	0	0%	5	45%	5	12%
Mirena IUD	0	0%	4	36%	4	10%
Depo-Provera	0	0%	2	18%	2	5%
None	30	100%	0	0%	30	73%
**Experienced a speculum-based pelvic exam**						
Yes	29	97%	11	100%	40	98%
No	1	3%	0	0%	1	2%
**Gravidity**						
Yes	27	90%	9	82%	36	88%
No	3	10%	2	18%	5	12%
**Parity**						
0	2	8%	0	0%	2	6%
One or more	24	92%	8	100%	32	94%
**Miscarriage***						
0	14	61%	8	100%	22	71%
One or more	9	39%	0	0%	9	29%
**Abortion**						
0	13	57%	4	44%	17	53%
One or more	10	43%	5	56%	15	47%
**Reproductive Surgeries**						
Yes ƚ	1	3%	0	0%	1	3%
No	28	97%	11	100%	39	98%
**Menopause**						
Yes	14	47%	2	18%	16	39%
No	15	50%	9	82%	24	59%
Unsure	1	3%	0	0%	1	2%

* Z-adjusted -2.01, $p<0.05$

ƚ bilateral tubal ligation

Supplementary Table 2. Influencing Physician and Women Characteristics

**Physician Characteristics**		
**For my healthcare, it is important for my physician to be the …**		
**Same gender as me**	**N**	**%**
Strongly disagree	5	12.2
Disagree	6	14.6
Neither agree nor disagree	13	31.7
Agree	10	24.4
Strongly agree	7	17.1
**Same religion/culture as me***		
Strongly disagree	8	20.0
Disagree	13	32.5
Neither agree nor disagree	18	45.0
Agree	1	2.5
Strongly agree	0	0
**Same race/ethnicity as me**		
Strongly disagree	11	27.5
Disagree	12	30.0
Neither agree nor disagree	10	25.0
Agree	3	7.5
Strongly agree	4	10.0
**Gender of the physician impacts my comfort with getting a pelvic exam§**		

**Physician Characteristics**		
Strongly disagree	13	31.7
Disagree	5	12.2
Neither agree nor disagree	9	22.0
Agree	7	17.1
Strongly agree	7	17.1
**Woman's characteristics**		
**I am uncomfortable/embarrassed to get a pelvic exam**		
Strongly disagree	17	42.5
Disagree	9	22.5
Neither agree nor disagree	8	20.0
Agree	4	10.0
Strongly agree	2	5.0
**I avoid a pelvic exam because of religious/cultural customs‡**		
Strongly disagree	29	72.5
Disagree	8	20.0
Neither agree nor disagree	3	7.5
Agree	0	0%
Strongly agree	0	0%

*Having the same physician religion/culture differs by age group, where younger women disagree more than older women (2.0 (0.9) vs. 2.6 (0.6), $p<0.05$).

§ Women with public health insurance disagreed that their comfort with the pelvic exam was dependent on the gender of the physician more than women with private insurance (2.4 (1.4) vs. 3.4 (1.4), $p<0.05$).
‡ Women with higher educational achievement disagreed that they would avoid a pelvic exam because of their own religious/cultural beliefs more than those with lower educational achievement (1.2 (0.4) vs. 1.6 (0.8), $p<0.05$).

<u>By physician characteristics</u>. Most women disagreed that their physician needed to be of the same race or ethnicity (82.5%) as themselves, and 97.5% of women disagreed that the physician needed to be of the same religion/culture as her. However, the Likert agreement scale frequencies showed even distribution in preferring a physician of her same gender for routine healthcare. Likewise, there was an even distribution across the Likert agreement scale for the "gender of the physician impacting her comfort with getting a pelvic exam."

The differences in women's preferences for specific physician characteristics were significant by their age and insurance status. Younger women disagreed more often that their physician needs to be of the same religion/culture as they are compared to older women (2.0 (0.9) vs. 2.6 (0.6), $p<0.05$). In addition, those women who had Medicare or Medicaid insurance disagreed more than women with private insurance that "the gender of the physician impacts my comfort level and willingness to participate in a pelvic exam" (2.4 (1.4) vs. 3.4 (1.4), $p<0.05$).

<u>By woman's characteristics</u>. Most women disagreed that they avoided a pelvic exam because of their own religious/cultural customs (92.5%). Women disagreed with their own sense of being uncomfortable or embarrassed by getting a pelvic exam more often than those who agreed (65% vs. 15%, $p<0.001$). However, women with higher educational achievement disagreed significantly more than those with lower educational achievement that they avoided a pelvic exam because of religious/cultural reasons (1.2 (0.4) vs. 1.6 (0.8), $p<0.05$).

Supplemental Table 3. Ranking of attributes of the last pelvic exam by demographic and physician characteristics

	**Education**	
	**Less than college education N=12**	**Some college or higher N=25**
My last pelvic exam was UNCOMFORTABLE*	2.3 (1.4)	3.8 (1.2)
	**Needs same-gender physician for a pelvic exam**	
	**Yes N=13**	**No N=16**
	mean (SD)	mean (SD)
My last pelvic exam was ANNOYING**	3.7 (1.4)	1.9 (1.0)
My last pelvic exam made me feel VULNERABLE*	3.5 (1.6)	1.7 (0.9)

Rankings are on a Likert scale from 1-5 where 1=strongly disagree, 5=strongly agree
*p<0.05, **p<0.01 corrected for multiple comparisons.

Supplementary Table 4. Menopausal status by age grouping

age	yes menopause	no menopause	unsure
30	0	2	0
31	0	1	0
33	0	3	0
34	0	1	0
35	0	2	0
37	0	1	0
38	0	3	0
39	0	1	0
41	0	2	0
42	0	2	0
43	0	1	0
44	0	1	0
45	0	1	0
46	0	1	0
48	1	0	0
49	1	0	0
51	2	0	0
52	0	1	0
54	2	0	0
56	1	0	0
57	1	0	0
59	2	1	0
60	1	0	0
61	2	0	1
63	2	0	0
65	1	0	0
